# Editorial for the Special Issue: “Antibiotic Use in Clinical Infection: How to Reinvent Old Molecules and How to Squeeze out New Ones”

**DOI:** 10.3390/antibiotics11050597

**Published:** 2022-04-29

**Authors:** Stefano Di Bella

**Affiliations:** Clinical Department of Medical, Surgical and Health Sciences, Trieste University, Piazzale Europa 1, 34127 Trieste, Italy; stefano932@gmail.com

The optimization of a number of strategies is required to overcome the problem of antibiotic resistance. A deep knowledge of bacterial resistance mechanisms, along with the correct and prompt use of old and new antibiotics and their combinations, are crucial when facing these instances of resistance. This Special Issue aimed to collect papers focusing on these strategies.

*Enterococcus faecium* is one of the strains of bacteria in the spotlight: vancomycin-resistant strains are spreading increasingly, causing significant clinical impact. Antibiotic options against vancomycin-resistant enterococci are limited; therefore, it is crucial to have a deep knowledge of this bug. Robert Weber et al. reported data on 101 *vanB*-positive *E. faecium* isolates, showing that dalbavancin (unlike against *vanA*) maintains a good in vitro activity with minimum inhibitory concentrations (MICs), similar to those of vancomycin-susceptible isolates, reaching values no higher than 0.125 mg/L. The authors, on the basis of the observed wild-type distribution, suggested a dalbavancin MIC of 0.25 mg/L as a tentative epidemiological cut-off value (ECOFF) for *E. faecium* [1].

With regard to “reinventing old molecules”, Tommaso Lupia et al. presented a literature review on temocillin, an old beta-lactam antibiotic with resurging clinical interest [2]. Temocillin has the pharmacokinetic/pharmacodynamic (PK/PD) advantages of beta-lactams antibiotics, and is active against ESBL, AmpC, and KPC-producing *Enterobacterales*. In addition, it is suitable for administration in continuous infusion and in elastomeric pumps, making it an appealing option for outpatient parenteral antibiotic therapy (OPAT). The majority of clinical data on temocillin are derived from urinary tract infections. Data regarding other infections are still limited by the fact that temocillin is only available in a few countries, but this may change in the next few years.

Another growing issue is the adoption of hemoadsorption systems for cytokine removal, especially in septic shock patients. This technique is providing interesting data; a strong consensus does not yet exist, but many centers are adopting this therapeutic strategy as a standard of care. It is important, in these situations, to keep in mind that hemoadsorption systems may eliminate part of the antibiotic/antifungal molecules. Giorgio Berlot et al. provided a comprehensive review on this topic, with indications on the amount of drugs cleared by hemoadsorption systems [3]. This will help clinicians in tailoring anti-infective doses in patients undergoing cytokine removal.

Regarding common clinical scenarios, infections in cirrhotic patients are commonly faced by internal medicine and infectious disease physicians. These patients commonly experience bacterial infections, and it is important to take into account their altered drug metabolism and the different fluid and protein distributions (ascites, hypoalbuminemia) that affect the PK/PD of antibacterials. Caterina Zoratti et al. have provided a paper that identifies all of the issues encountered in cirrhotic patients, and suggests ways to tackle them when adequate antibiotics are needed [4].

Alberto Maraolo et al. have contributed a meta-analysis to the Special Issue, comparing daptomycin with vancomycin against methicillin-resistant *Staphylococcus aureus* invasive infections (bloodstream infections and endocarditis). The authors evaluated 8 studies including a total of 1226 patients: 554 received daptomycin and 672 received vancomycin. They found no difference in terms of overall mortality, but a significant reduction in clinical failure (OR 0.58, 95% CI 0.38–0.89) and in adverse events (OR 0.15, 95% CI 0.06–0.36) for those receiving daptomycin [5].

Among encountered bacterial resistances, metallo beta-lactamases (MBL) enzymes represent a problem with few options. Rather than waiting for new solutions, it was necessary to “reinvent” old drugs such as aztreonam, a monobactam antibiotic that is not inhibited by MBL enzymes. However, since approximately half of the MBL-producing strains that co-produce ESBL are capable of hydrolyzing aztreonam, the addition of avibactam could protect aztreonam, allowing it to act. Carola Mauri et al. presented a systematic review of in vivo studies and clinical cases of patients treated with an aztreonam/avibactam combination. Clinical data were available for ~100 patients, and mortality for bloodstream infections was “only” 19% in those treated with aztreonam/avibactam, rendering this a promising strategy against these difficult-to-treat infections [6].

Finally, Davide Carcione et al. provided a review on old and new beta-lactamase inhibitors in terms of chemical structure, mechanisms of action, spectrum of activity, clinical use, and PK/PD properties. Clavulanic acid, sulbactam, tazobactam, avibactam, relebactam, vaborbactam, zidebactam and nacubactam were comprehensively reviewed [7].

It is important to keep in mind that using the above-mentioned strategies in an appropriate manner is crucial to preventing them from becoming useless.

I am proud of this Special Issue and of its pragmatic perspective. I believe it can be used both for research purposes and for consultation for the management of insidious clinical situations.
